# Historical Trends and Implications of the Sex Ratio at Birth in Georgia, 1926–2024

**DOI:** 10.21203/rs.3.rs-10469421/v1

**Published:** 2026-08-27

**Authors:** Khatia Otarashvili, Nino Nazirishvili, Eka Paatashvili, Ali Mirzazadeh

**Affiliations:** 1 Faculty of Natural Sciences and Medicine, Department of Public Health, Ilia State University, Tbilisi, Georgia; 2 Institute for Global Health Sciences, University of California, San Francisco, USA; 3 Faculty of Medicine, European University

## Abstract

**Background:**

The sex ratio at birth (SRB) is a key demographic indicator, typically stable within a natural range of 102–106 male births per 100 female births. Deviations from this range may reflect biological or non-natural influences, including sex-selective practices. Despite elevated SRB levels in the South Caucasus, Georgia lacks a country-specific historical baseline.

**Objective:**

This study reconstructs long-term SRB trends in Georgia from 1926 to 2024, estimates the country-specific natural SRB baseline, and examines Georgia’s trajectory within the broader South Caucasus context.

**Methods:**

We analyzed national demographic data from Georgia, combining historical census data, civil registration records, and United Nations estimates. For 1926–1950, SRB was reconstructed using the sex ratio among children aged 0–4 years and a correction factor derived from 1970–1980 data. Trends were compared with Armenia and Azerbaijan for 1950–2020.

**Results:**

SRB in Georgia remained within the natural range until the late 1980s, generally around 105–106 males per 100 females, with a minimum of 101.2 in 1979. It increased sharply in the 1990s, reaching approximately 111 in the early 2000s, followed by a gradual decline but remained above natural levels through 2020. Armenia and Azerbaijan showed higher peaks, reaching approximately 117.

**Conclusions:**

This study provides the first long-term analysis of the sex ratio at birth (SRB) in Georgia and establishes a country-specific historical baseline, offering a robust benchmark for identifying deviations from the expected natural range.

**Contribution:**

This study consolidates a century-long record of the sex ratio at birth (SRB) for Georgia and establishes a demographic baseline for assessing gender imbalances and regional disparities.

## Introduction

The sex ratio at birth (SRB), or secondary sex ratio, is a demographic indicator defined as the ratio of male to female children born alive, it is usually expressed as the number of live births of boys per 100 girls. Under natural conditions, SRB is relatively stable, generally ranging from 103–106 boys per 100 girls (United Nations Secretariat 1998; Visaria 1971), with an average value of approximately 105, representing the expected biological range (Bongaarts 2013; Dyson 2012; United Nations 2011; James and Grech 2017; Mathews and Hamilton 2005). A sex ratio at birth that rises significantly above 106 or falls well below 102 often indicates non-natural influences or external interventions, such as sex-selective abortion or other socio-cultural influences (World Health Organization 2021; UNFPA 2012).

The SRB is not only a biological indicator but also an important marker of population dynamics, reflecting interactions between reproductive behavior, social norms, gender preferences, and demographic processes (Bernard et al. 2022; Guilmoto 2009; Guilmoto 2012; Das Gupta 2005). Persistent deviations from the expected SRB range may have long-term consequences for population structure, marriage markets, family formation patterns, and gender balance within societies (Guilmoto 2009; Guilmoto 2012; Das Gupta 2005). In this context, monitoring SRB trends provides valuable information for understanding demographic transitions and identifying populations affected by gender-biased reproductive practices.

SRB may decrease due to maternal physiological state and environmental factors (Fontanesi et al. 2024; Schacht, Tharp, and Smith 2019; Fukuda et al. 2020). Maternal stress during pregnancy, which can be caused by individual factors such as a significant loss, anxiety, or depression (Grech 2018; Hansen et al. 1999; Schnettler and Klüsener 2014), or by environmental factors such as wars and conflicts (Grech 2014; James 2009), terrorist attacks (Masukume, Chibueze, and Ray 2017), or natural disasters (Saadat 2008). Maternal stress is associated with low birth weight, genetic defects, short gestational age, lower gestational age, increase the risk for preterm birth, and neonatal mortality (Ding et al. 2021; Heron 2018; Kim et al. 2017; Parayiwa and Behie 2018; Suzuki et al. 2016). The stress in the second and third trimesters of pregnancy are particularly associated with these risks (Di Renzo et al. 2007; Obel et al. 2007).

A male fetus is biologically more vulnerable and more sensitive to maternal stress and negative environmental factors than a female fetus (Kirchengast and Hartmann 2009; Abdoli 2022), therefor fetal loss and premature death are more common in boys, which can naturally be reflected in the reduced SRB (having less boys born) (McCarthy 2019).

Social public stress such as terrorist acts, war, economic crises, and natural disasters can reduce the SRB by about 1–3 points, with the effect typically appearing temporarily within 3–6 months after the event (Catalano et al. 2006; Bruckner and Catalano 2010; Grech 2015; Trivers and Willard 1973; D’Alfonso et al. 2012). Natural variation also occurs between ethnic and racial groups, with differences of about 2–4 points (Mathews and Hamilton 2005; Grech 2015). According to the Trivers-Willard hypothesis, parental physical and social stability influences birth outcomes, with better resource conditions associated with higher male birth rates, while stressful conditions may reduce SRB (Trivers and Willard 1973; James and Grech 2017; Wu 2021) to below 102. Environmental and geographic factors also contribute to variation, typically within the natural range of 102–106 (James 1996; Grech 2015). Altitude may further influence SRB, as mountainous regions have been associated with decreases of about 1–2 points (Grunebaum and Chervenak 2016).

The SRB natural variation exists across ethnic groups and regions. In the United States, SRB tends to be lowest among non-Hispanic Black populations (about 102–104), intermediate among Hispanics (about 103–105) and non-Hispanic Whites (about 104–106), and highest among non-Hispanic Asians (about 105–107). Large U.S. birth record analyses show similar patterns, with Asian or Pacific Islander births having the highest male-to-female ratios, Whites intermediate, and Black or African American births the lowest (Mathews and Hamilton 2005).

SRB is a key demographic indicator that reflects not only biological norms but also sociocultural practices such as sex-selective abortion (Chao et al. 2019; Davis, Gottlieb, and Stampnitzky 1998). However, Georgia remains understudied in the global SRB literature, despite evidence of gender imbalances in comparable contexts such as Armenia and Azerbaijan. Although previous studies have described SRB patterns in Georgia, they have primarily relied on comparisons with international reference values or limited contemporary periods. To date, no study has established a Georgia-specific historical baseline of natural SRB or reconstructed long-term trends covering nearly a century, particularly for the period before the availability of reliable birth registration data.

An analysis of Georgia’s historical data (1926–2024) addresses this gap by documenting long-term SRB trends, providing an estimate of a national natural baseline, and enabling comparisons within the South Caucasus and the broader post-Soviet region. Given Georgia’s historically diverse population structure, including substantial Armenian and Azerbaijani ethnic minority communities, comparison with neighboring South Caucasus countries provides an important contextual framework for interpreting regional demographic patterns. However, these comparisons should not be interpreted as direct evidence of ethnic-specific SRB differences, as the present study relies on national-level aggregated data. Instead, they allow assessment of whether observed trends in Georgia correspond to broader regional demographic transformations or reflect country-specific trajectories. Such evidence is essential for understanding shared cultural, economic, and political influences, while also informing regional comparisons, unified policy planning, and national public health strategies aimed at preventing sex discrimination and strengthening reproductive and family policies.

Long-term monitoring of SRB trends provides important evidence for distinguishing biological variation from demographic, social, and environmental influences affecting sex composition at birth (Fontanesi et al. 2024; Chao et al. 2019). Therefore, this study aims to provide a century-long assessment of the sex ratio at birth (SRB) in Georgia from 1926 to 2024, including the reconstruction of previously undocumented trends for 1926–1950, to establish a historical baseline for the natural SRB and identify temporal and regional sex-ratio imbalances.

## Methods

This study used a retrospective population-based demographic design to reconstruct long-term trends in the sex ratio at birth (SRB) in Georgia from 1926 to 2024 using aggregated national-level demographic data. The study combined direct estimates derived from civil registration systems with historical demographic reconstruction methods to establish a country-specific baseline SRB and evaluate deviations from the expected biological range over time.

### Data Sources and Study Period

We used nationally representative demographic data from the National Statistics Office of Georgia (GeoStat) and the United Nations Population Division’s World Population Prospects (WPP 2019). The dataset included historical population censuses (1926–1980), annual live birth statistics (1960–2024), population age structures, and mortality indicators required for demographic adjustments. Specifically, the analysis incorporated national-level counts of male and female live births, census-based population age distributions, and infant and under-five mortality data to perform sex-specific demographic corrections (United Nations 2019; GeoStat 2024).

GeoStat data were used as the primary national source because they provide the longest available demographic serios for Georgia, including historical population structures, registered births, and mortality indicators. These data enable the reconstruction of SRB during periods when direct birth registration by sex was incomplete or unavailable.

We used available data from multiple sources (Table 1), including historical demographic datasets (1926–1980), contemporary civil registration records (1950–2020; 1960–2022; 1994–2024) and Under-five mortality rate (1950–2025), to provide an estimate of Georgia’s natural biological sex ratio at birth. Historical data are crucial for assessing the natural level of SRB during periods when sex-selective abortion was rare or not exist (United Nations 2019; GeoStat 2024).

In addition to SRB, we also included the sex ratio of the population aged 0–4, which was available for Georgia from 1926–1980 (Geostat). This allowed to estimate a **modelled sex ratio at birth (SRB)** using the observed sex ratio among children aged 0–4 years (SR0_4) and the average correction factor (CF_avg) when direct birth records to calculate SRB were unavailable between 1926 and 1950 (GeoStat 2024).

The reliable, continuous, and internationally comparable SRB data for Georgia, Armenia, and Azerbaijan were available only from around 1950 to 2020, and so our analysis of SRB started in the mid-twentieth century. The latest validated SRB data were available for 2020; therefore, the trend analysis covered the period from 1950 to 2020 (United Nations 2019); and also, sex ratio data at birth were used separately for Georgia from 1960, 1965, 1969 to 2024 (GeoStat 2024).

The study period was divided according to data availability. Historical reconstruction was performed for 1926–1950, when direct SRB estimates were unavailable, while observed SRB trends were analyzed for periods with available birth registration data. For international comparison, harmonized SRB estimates from the United Nations World Population Prospects were used for Georgia, Armenia, and Azerbaijan from 1950 to 2020.

### Historical reconstruction of natural Sex Ratio at Birth

Complete birth registration data disaggregated by sex were unavailable for the earliest decades of the study period. Therefore, the estimated natural SRB for 1926–1950 was reconstructed indirectly using the sex composition of children aged 0–4 years. Indirect demographic reconstruction methods based on age-sex population structures and mortality adjustments are widely used in historical demography when direct vital registration data are incomplete or unavailable. These approaches allow researchers to estimate past demographic indicators by correcting observed population structures for differential survival patterns (Shryock and Siegel, 1976; Coale and Demeny, 1983; Preston, Heuveline, and Guillot, 2001; United Nations, 1983; United Nations, 2019).

The sex ratio among children aged 0–4 years (SR_0–4_) was calculated as the number of males aged 0–4 years divided by the number of females of the same age group and multiplied by 100. Because mortality risks during early childhood differ by sex, particularly due to higher male mortality in infancy and early childhood, the observed sex composition of surviving children may underestimate the original sex ratio at birth. Therefore, a correction factor based on differential survival patterns was applied to reconstruct the expected SRB. Similar correction approaches using mortality-adjusted age-sex structures have been applied in historical population studies and demographic reconstruction (Coale and Demeny, 1983; Preston, Heuveline, and Guillot, 2001; United Nations, 2019).

This reconstruction allowed the establishment of a continuous long-term SRB trajectory for Georgia, covering approximately a century of demographic change, and provided a historical baseline against which contemporary deviations in SRB could be evaluated.

### Data Reliability

To ensure that long-term SRB trends in Georgia were analyzed using reliable inputs, contextual information regarding historical registration practices and reproductive health policies was obtained through consultations with key Georgian stakeholders, including a public health expert specializing in reproductive health policy, a representative of the Ministry of Health, and a representative of the birth registration department of the NCDC.

These consultations were used to support interpretation of demographic trends and identify potential changes in registration systems that could affect comparability between historical and contemporary data.

### Data analysis

The SRB was calculated by dividing the number of boys born alive each year by the number of girls and multiplying the result by 100. The resulting figures were included in the analysis for the entire country. We analyzed the population data for 0–4 years old for 1926 to provide an approximate picture of the situation of the SRB in years before 1950 where sex at birth was not reported.

Temporal trends were assessed using descriptive analysis of annual SRB estimates and comparisons across historical periods. Reconstructed SRB estimates were compared with observed SRB values during overlapping periods to evaluate consistency between indirect and direct estimates.

Sex-specific under-five mortality probabilities were not available for the period 1926–1950. Consequently, direct estimation of sex-specific survival probabilities (S_b for boys and S_g for girls) and deaths at birth (DeathsAtBirth_Boy, DeathsAtBirth_Girl) was not feasible. To address this limitation, we estimated the sex ratio at birth (SRB) indirectly using the observed sex ratio among children aged 0–4 years ($SR_{0-4}$), adjusted by a correction factor (CF). This factor accounts for differential survival between boys and girls in early childhood. The modelled SRB was calculated as:

(1) $$SRB=SR_{0-4}\times CF_{\mathrm{avg}}$$

The correction factor (CF) was derived from a period with reliable data on both SRB and $SR_{0-4}$. Given that our dataset spans nearly a century (1926–2024) and that sex-specific under-five mortality in Georgia varies over time, we selected an intermediate and relatively stable period (1970–1980) for calibration.

The 1970–1980 period was selected as the calibration period for several methodological reasons. First, this interval represented the earliest period with sufficiently reliable sex-disaggregated mortality data and observed SRB estimates available simultaneously, allowing the empirical calculation of the correction factor. Second, it represented an intermediate period within the overall study timeframe, reducing the risk of applying contemporary mortality patterns to historical populations. Third, this period preceded the substantial improvements in child survival observed in later decades. Therefore, using more recent mortality coefficients could introduce systematic bias when reconstructing earlier SRB estimates. The average correction factor derived from 1970–1980 was therefore considered the most appropriate available calibration parameter for estimating historical SRB during 1926–1950.

For each year in this interval, CF was computed as:

(2) $$CF=\frac{SRB}{SR_{0-4}}$$

The average correction factor (CF_avg) across 1970–1980 was then applied to estimate SRB for the earlier period (1926–1950), assuming that the relationship between SRB and $SR_{0-4}$ during the calibration period provides a reasonable approximation in the absence of direct data. Using the estimated SRB and overall under-five mortality ($q_{5}$), we derived sex-specific survival probabilities under the assumption that the average survival probability (S_avg) is shared between sexes and adjusted proportionally using CF:

(3) $$S_{avg}=1-q_{5}$$

(4) $$S_{b}=\frac{2\times S_{avg}}{1+CF},S_{g}=CF\times S_{b}$$

Finally, deaths at birth were calculated as the complement of survival probabilities:

(5) $${\mathrm{DeathsAtBirth}}_{\mathrm{Boy}}=1-S_{b}$$

(6) $${\mathrm{DeathsAtBirth}}_{\mathrm{Girl}}=1-S_{g}$$

This approach is methodologically supported in the historical demographic literature (Hill & Upchurch, 1995; United Nations, 2019), which shows that although absolute under-five mortality was unknown, the relative male disadvantage in early childhood survival has been consistently observed across populations and historical periods.

### Comparative analysis:

For comparative purposes, the SRB trends in Georgia were compared with those of Armenia and Azerbaijan over the period 1950 to 2020. This allowed us to contextualize Georgia’s SRB within the broader South Caucasus region, identify regional patterns, and assess similarities or divergences in demographic trends across neighboring populations.

The comparative approach was used because Georgia shares historical and demographic characteristics with other South Caucasian countries, while differences in SRB trajectories may reflect country-specific reproductive and social patterns. Furthermore, Georgia has a historically diverse population that includes Armenian and Azerbaijani ethnic communities, which share cultural and demographic links with neighboring Armenia and Azerbaijan. Therefore, comparison with these countries provides an additional regional framework for interpreting SRB patterns and exploring broader sociocultural and demographic similarities, while recognizing that the present study does not directly assess ethnic-specific differences within Georgia.

Data for Armenia and Azerbaijan were obtained from the United Nations World Population Prospects (United Nations, 2019) and compared with available national statistical records where applicable to ensure consistency and comparability with Georgian SRB data.

### Data eligibility criteria:

Data were included in the analysis if they met the following criteria: (1) nationally representative demographic data for Georgia, Armenia, or Azerbaijan; (2) availability of annual sex ratio at birth (SRB) or sex ratio among children aged 0 to 4; and (3) consistency with official statistical sources, including the National Statistics Office of Georgia and the United Nations World Population Prospects database. Data were excluded when records were incomplete, inconsistent across sources, or lacked sufficient information to calculate or approximate the sex ratio indicators required for the analysis.

## Results:

**Figure 1.**
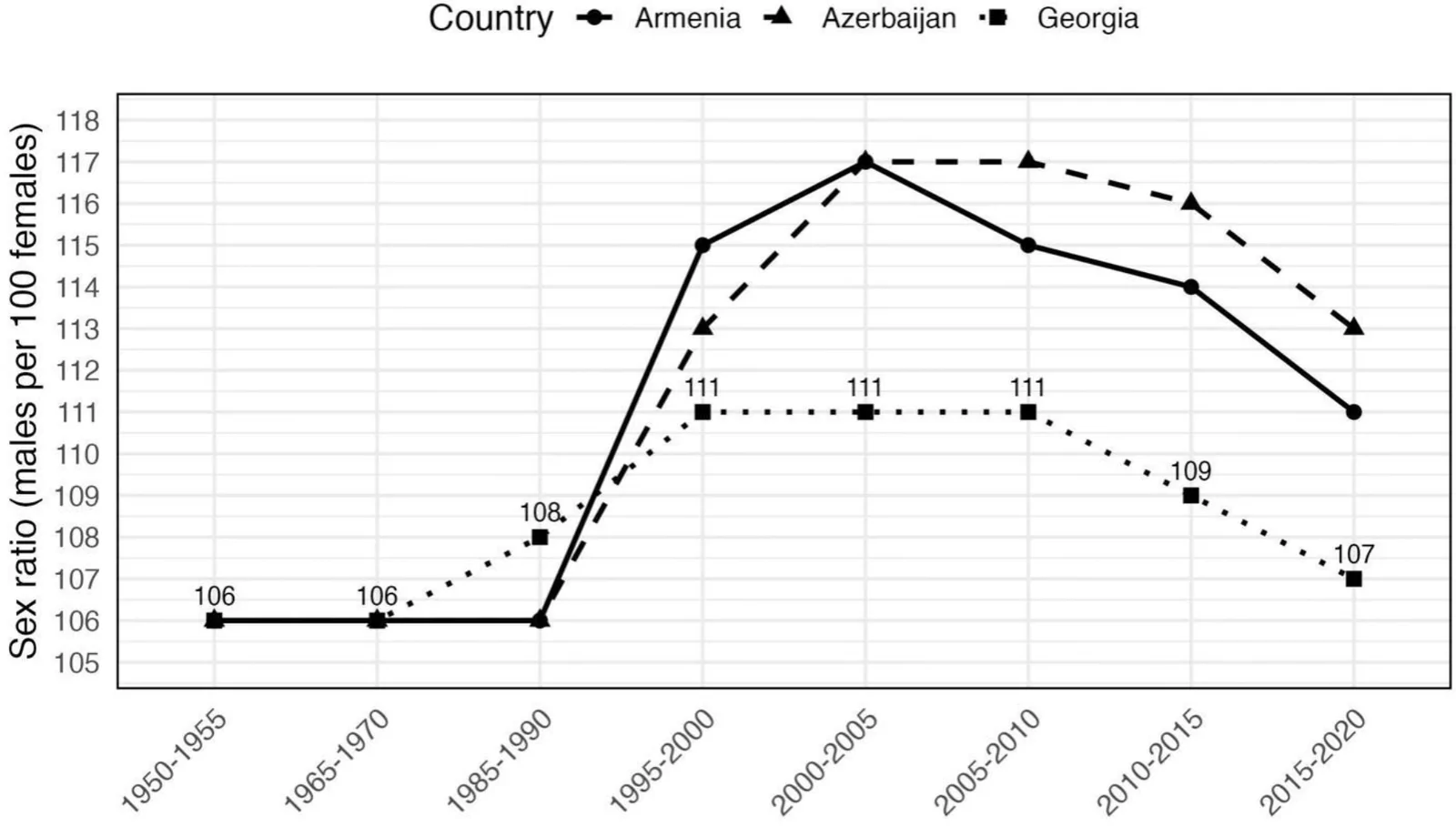
Trends in Sex Ratio at Birth (Males per 100 Females) in three countries in the South Caucasus region, 1950 to 2020

Figure 1 shows distinct but broadly similar trends in the SRB across the three South Caucasus countries between 1950 and 2020. During the early period (1950s-1980s), SRB levels in Armenia, Azerbaijan, and Georgia remained close to the biological norm (around 106 males per 100 females). A sharp divergence emerged in the 1990s, with all three countries experiencing marked increases in SRB, peaking in the early 2000s. The rise was most pronounced in Armenia and Azerbaijan, where SRB reached approximately 117 males per 100 females, while Georgia showed a more moderate peak of about 111. After 2005, SRB levels declined in all countries, though they remained above natural levels through 2020, particularly in Azerbaijan.

**Figure 2.**
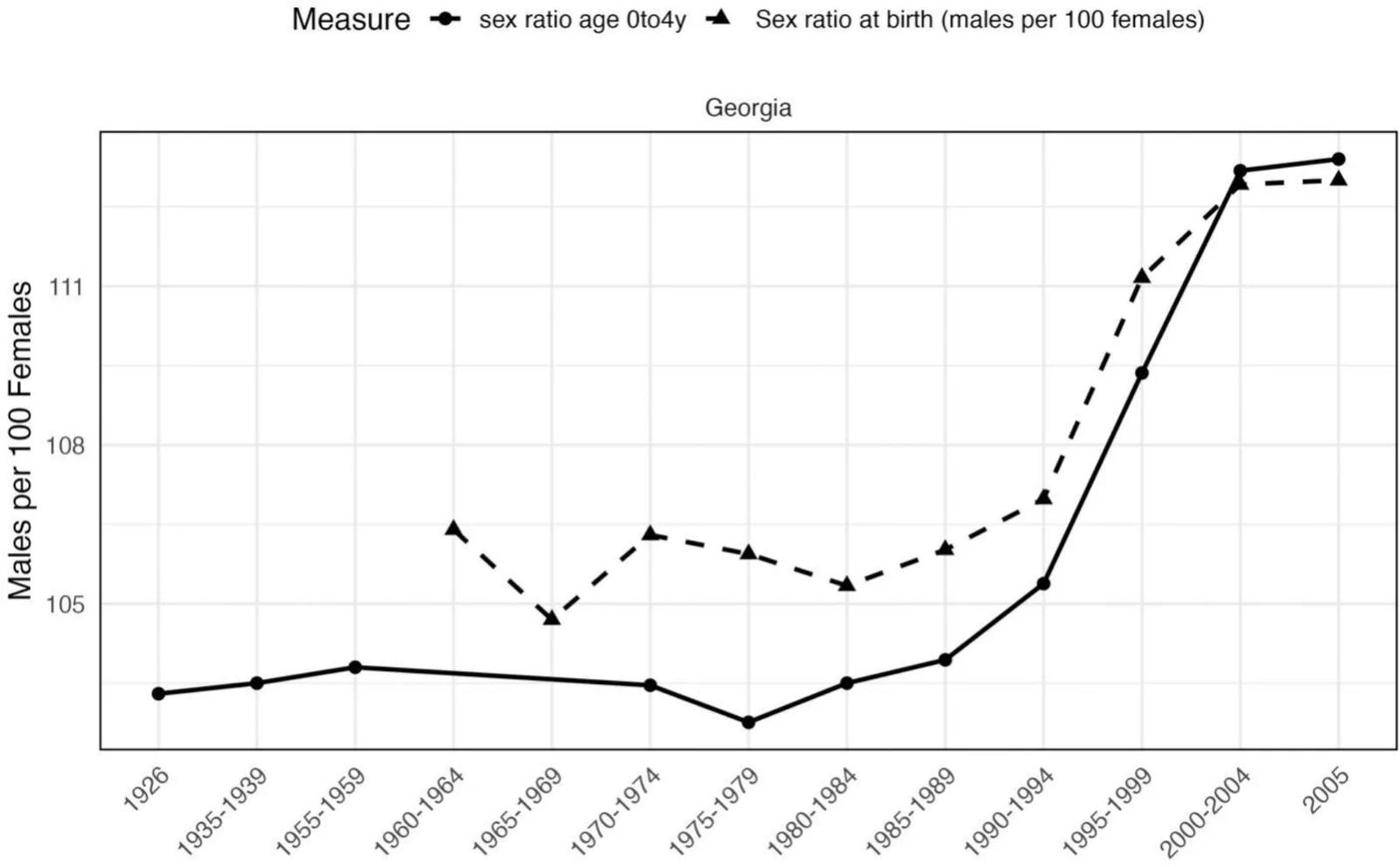
Sex Ratios in Georgia at Birth and Among Children Aged 0 to 4 Years, 1926 to 2005

Figure 2 shows long-term trends in Georgia’s SRB and the sex ratio among children aged 0–4 years from 1926 to 2005. For much of the twentieth century, both measures remained close to the biological norm (around 106), with only modest fluctuations and a consistent pattern in which the SRB was slightly higher than the sex ratio at ages 0–4. Beginning in the early 1990s, a pronounced increase is evident in both indicators, signaling a departure from natural levels. The rise was sharper and appeared earlier in the SRB, followed closely by a corresponding increase in the sex ratio among young children, reflecting the demographic impact of elevated SRB on cohort composition. By the early 2000s, both measures reached similarly high levels (around 112–113 males per 100 females), showing a substantial shift toward gender imbalance during this period.

A supplementary figure (Figure S1) shows that from 1926 through the late 1980s, both the sex ratio at birth and the sex ratio among children aged 0–4 in Georgia remained close to biological norms with only minor year-to-year fluctuations. Beginning in the early 1990s, both indicators increased sharply, with the rise in the sex ratio at birth preceding and closely mirrored by increases among children aged 0–4. In 1979, the SRB reduced to its minimum of 101.2.

## Discussion:

Our study examined long-term trends in the sex ratio at birth (SRB) in Georgia over nearly a century and established a country-specific historical baseline for interpreting deviations from expected levels. The analysis identified an estimated natural SRB level of approximately 105 male births per 100 female births. Throughout most of the twentieth century, SRB in Georgia remained stable and within the expected biological range, with only minor fluctuations. A substantial change became evident in the early 1990s, when SRB increased rapidly, reaching its highest level in the early 2000s, followed by a gradual decline. However, despite this decline, SRB has not yet returned to the levels observed before the transition period. Comparisons with Armenia and Azerbaijan demonstrated similar regional patterns, although the magnitude of the increase was considerably greater in these countries. The parallel changes observed in SRB and the sex ratio among children aged 0–4 years further indicate the demographic consequences of altered birth patterns over time. By extending the available evidence beyond the period of contemporary birth registration, this study provides a historical demographic framework for interpreting recent SRB changes in Georgia. Overall, these findings provide the first comprehensive historical reconstruction of SRB trends in Georgia and demonstrate a transition from long-term demographic stability to elevated sex ratios following the post-Soviet period.

The stability of SRB in Georgia until the 1990s suggests that birth sex composition during this period was primarily influenced by biological mechanisms rather than observable population-level deviations associated with reproductive selection practices. The consistency of SRB within the expected biological range, together with the similar stability observed in the sex ratio among children aged 0–4 years, indicates that systematic sex selection was unlikely to have produced measurable demographic effects at the national during this period. This observation is consistent with the limited availability of prenatal sex determination technologies and the reproductive context of the Soviet period, when opportunities for sex-based reproductive decision-making were considerably restricted. Nevertheless, existing preferences regarding child sex may have been present, but the prevailing institutional, technological, and socioeconomic conditions likely limited their translation into practices capable of altering the overall sex composition at birth (Das Gupta, 2005; Guilmoto, 2009).

The sharp increase in SRB observed since the early 1990s is likely associated with the interaction of technological, socioeconomic, and sociocultural changes that occurred during the post-Soviet transition. The expanding availability of prenatal diagnostic technologies, particularly ultrasound, enabled earlier identification of fetal sex and created new opportunities for sex-based reproductive decision-making. Concurrently, the socioeconomic instability of the transition period, declining fertility rates, and the shift toward smaller family sizes may have increased the importance of achieving a preferred family composition, including son preference in some contexts.

Recent evidence from Georgia further suggests that reproductive health policies and regulatory changes may have influenced SRB patterns during the post-transition period. A study analyzing 920,803 live births between 2005 and 2022 demonstrated a gradual decline in the male-to-female birth ratio following major changes in abortion regulation and fetal sex disclosure policies, with the most pronounced reductions observed among higher-order births and regions that previously exhibited elevated SRB levels (Otarashvili et al., 2026). Although these findings do not establish direct causality at the individual level, they provide additional evidence that institutional and regulatory contexts may modify the extent to which existing preferences regarding child sex are reflected in population-level birth patterns.

Although the present ecological design cannot directly determine individual-level motivations or reproductive decisions, the temporal association between increased SRB and major demographic and technological transitions suggests that these factors may have contribute to the observed changes. Under these changing conditions, previously existing preferences regarding child sex may have become more likely to influence reproductive outcomes. The earlier and more pronounced increase in SRB compared with changes in the sex ratio among children aged 0–4 years suggests that the initial alteration occurred at the level of births and was subsequently reflected in the demographic structure of younger cohorts. These findings are consistent with regional and international evidence indicating that elevated SRB patterns often emerge in contexts characterized by the combination of access to prenatal sex determination technologies, declining fertility, and persistent gender preferences (Guilmoto, 2009; Duthé et al., 2012; United Nations Population Fund, 2012).

A comparison of Georgia with Armenia and Azerbaijan demonstrates both shared regional patterns and important differences in the magnitude of SRB changes across countries. In all three South Caucasus countries, SRB increased substantially after the collapse of the Soviet Union, suggesting the influence of common structural transformations, including socioeconomic transition, declining fertility, and increased access to prenatal diagnostic technologies. However, the higher SRB levels observed in Armenia and Azerbaijan indicate a greater degree of demographic imbalance compared with Georgia, which may reflect differences in the prevalence and intensity of son preference and related reproductive practices.

This regional comparison is particularly relevant for understanding the Georgian context, given the country’s demographic diversity and the presence of Armenian and Azerbaijani ethnic communities. Shared historical, cultural, and social characteristics within the South Caucasus provide an important framework for interpreting broader regional patterns while recognizing potential internal variations. Nevertheless, Georgia’s comparatively lower peak SRB may suggest either weaker son preference or a lower likelihood that such preferences were translated into reproductive behaviors affecting birth outcomes. Overall, the persistence of elevated SRB levels across all three countries highlights the regional dimension of sex ratio imbalance while also emphasizing the importance of country-specific social and demographic factors (Meslé et al., 2007; Guilmoto, 2012; World Bank, 2019).

These findings have important implications for demographic surveillance and reproductive health policy in Georgia. Continued monitoring of SRB may help identify population groups at increased risk of sex imbalance and support evidence-based interventions aimed at promoting gender equity and preventing sex-selective practices.

### Limitations

Several limitations should be considered when interpreting the findings of this study. First, the analysis is based on aggregated population-level data and therefore cannot directly establish links between observed SRB patterns and individual-level reproductive behaviors, including sex-selective abortion. The findings should therefore be interpreted as describing demographic patters at the population level rather than individual reproductive decisions or motivatons.

Second, the reconstruction of SRB for the earlier period (1926–1950) relied on indirect estimation using the sex ratio among children aged 0–4 years and a correction factor derived from a later calibration period with more reliable data availability. Although this approach is consistent with established demographic methods, it assumes that the relationship between sex-specific survival patterns and SRB remained relatively stable over time, which may not fully apply across the entire historical period.

In particular, the application of the correction factor derive from the 1970–1980 calibration period represents an important methodological assumption. Although this period was selected because it provided the most reliable combination of sex-specific mortality indicators and observed SRB data within the available historical timeframe, changes in healthcare access, child survival, and demographic conditions over the twentieth century may have affected the magnitude of sex-specific mortality differences. Therefore, reconstructed estimates for earlier decades should be interpreted as the most plausible demographic approximation rather than direct observations.

Third, potential limitations in data quality, particularly for earlier historical records and periods of political transition, may have influenced the precision of the estimates. Historical demographic data may be affected by incomplete registration, administrative changes, and variations in data collection practices, especially during the first half of the twentieth century. To minimize these effects, multiple independent data sources were used and reconstructed estimates were compared with available observed SRB trends where possible.

Furthermore, the study does not account for internal population heterogeneity, including regional, ethnic, and socioeconomic differences, or the potential influence of migration patterns on SRB trends.

Despite these limitations, the reconstruction approach applied in this study follows established methods in historical demography and population studies. The use of multiple complementary data sources and comparison with international demographic estimates provides additional confidence in the overall interpretation of long-term SRB patterns in Georgia.

Given these limitations, the findings should be interpreted primarily as evidence of population-level demographic changes rather than direct evidence of individual reproductive decision-making. Further research incorporating individual-level data, regional analyses, and qualitative approaches is needed to better understand the underlying social and behavioral mechanisms contributing to SRB variation in Georgia.

## Conclusion

This study demonstrates a substantial long-term transformation of the sex ratio at birth in Georgia, from a period of relative demographic stability within the expected biological range to the emergence of elevated SRB levels during the post-Soviet period. The observed trends are consistent with broader demographic patterns identified across the South Caucasus while also highlighting differences in the magnitude of change between countries.

The close correspondence between SRB trends and the sex ratio among children aged 0–4 years indicates that changes in birth patterns have resulted in measurable and persistent demographic consequences. The Georgia-specific historical baseline of approximately 105 male births per 100 female births provides an important reference point for future assessments of gender imbalance and deviations from expected levels. By establishing this historical baseline, the study provides a framework for distinguishing long-term biological patterns from more recent demographic deviations associated with changing reproductive, social and policy contexts.

Further research should investigate regional and population subgroup variations within Georgia to better understand the internal dynamics of SRB changes and the social, cultural, and demographic factors associated with these patterns. Future studies integrating individual-level reproductive data and qualitative evidence may provide further insight into the mechanisms contributing to SRB variation in Georgia. Such analyses would contribute to a more comprehensive understanding of sex ratio imbalance and support the development of targeted policies addressing its underlying determinants.

## Supplementary Material

Supplement 1

Supplementary Files

This is a list of supplementary files associated with this preprint. Click to download.

• DataKhatiaPaper11March2026.xlsx

## Figures and Tables

**Table 1.** Data sources and demographic indicators used for reconstruction of sex ratio at birth in Georgia, 1926–2024

Data source	Period covered	Variables used	Purpose in analysis
National population censuses (GeoStat)	1926–1980	Population by sex and age groups, including children aged 0–4 years	Reconstruction of historical SRB for periods without direct birth registration data
Civil registration and vital statistics (GeoStat)	1960–2024	Annual live births by sex	Direct calculation of observed SRB trends in Georgia
United Nations World Population Prospects (WPP 2019)	1950–2020	SRB estimates, population structure, mortality indicators	Validation of national estimates and comparative analysis with Armenia and Azerbaijan
Under-five mortality estimates (UN WPP)	1950–2025	Overall under-five mortality probability (q₅)	Estimation of sex-specific survival probabilities and correction factors
Regional demographic data (UN WPP 2019)	1950–2020	SRB trends in Armenia and Azerbaijan	Comparative analysis of South Caucasus SRB patterns

## Data Availability

The demographic data used in this study were obtained from the National Statistics Office of Georgia (GeoStat) and the United Nations Department of Economic and Social Affairs, Population Division (World Population Prospects). The original data sources are publicly available through the official GeoStat and United Nations databases. To facilitate transparency and reproducibility, the datasets and supporting documentation used in this study have been provided as Supplementary Materials. These materials include the historical demographic dataset for Georgia (1926–2024), comparative SRB datasets for Armenia and Azerbaijan (1950–2020), processed analytical datasets, variable definitions, and documentation of the calculations and demographic adjustment procedures used for the reconstruction of historical sex ratio at birth (SRB) estimates. All datasets used in this study are aggregated population-level demographic data and contain no personally identifiable information. The analytical procedures and derived datasets are provided solely for the purpose of enabling verification and replication of the reported findings.
