# Supplementary material for: Historical Trends and Implications of the Sex Ratio at Birth in Georgia, 1926–2024
Source: Res Sq. 2026 Aug 27:rs.3.rs-10469421. Preprint. [Version 1] doi: 10.21203/rs.3.rs-10469421/v1 (PMC13532718; doi:10.21203/rs.3.rs-10469421/v1)
Supplement: Supplementary file 1 [file NIHPPRS10469421V1-supplement-1.pdf]

Supplament figure:

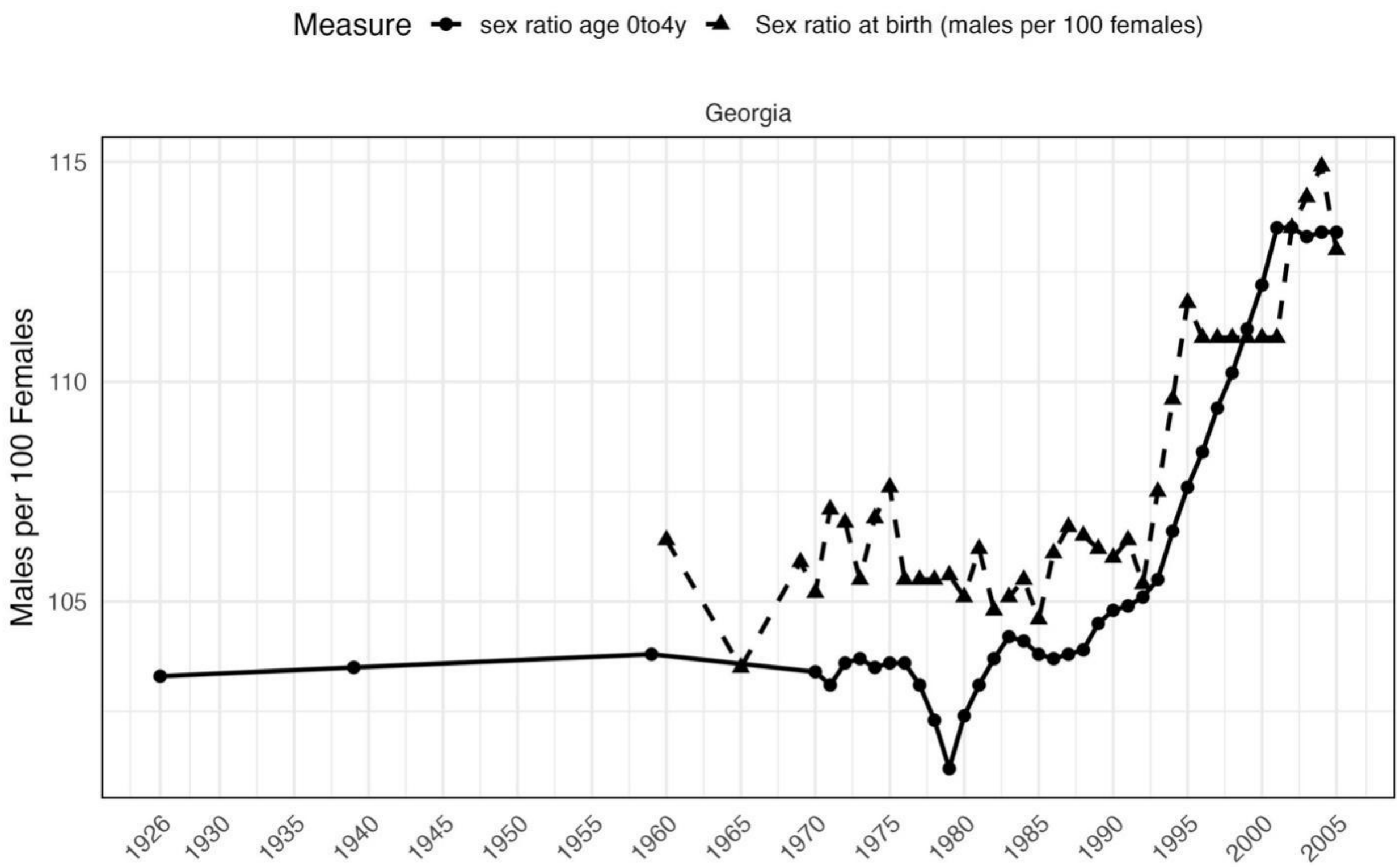

Figure 1s. Sex Ratio Trends in Georgia, At Birth and Among Children Aged 0 to 4 Years, 1926 to 2005
